# Targeting FGFRs by pemigatinib induces G1 phase cell cycle arrest, cellular stress and upregulation of tumor suppressor microRNAs

**DOI:** 10.1186/s12967-023-04450-7

**Published:** 2023-09-15

**Authors:** Angelica Pace, Fabio Scirocchi, Chiara Napoletano, Ilaria Grazia Zizzari, Agnese Po, Francesca Megiorni, Angela Asquino, Paola Pontecorvi, Hassan Rahimi, Cinzia Marchese, Elisabetta Ferretti, Marianna Nuti, Aurelia Rughetti

**Affiliations:** 1 Department of Experimental Medicine, “Sapienza” University of Rome, Rome, Italy; 2 Department of Molecular Medicine, “Sapienza” University of Rome, Rome, Italy

**Keywords:** Pemigatinib, FGFR, TKI, Cell cycle arrest, Senescence, Apoptosis, ROS, Calreticulin, Cellular stress, miRNA

## Abstract

**Background:**

Fibroblast growth factor receptor (FGFR) gene family alterations are found in several cancers, indicating their importance as potential therapeutic targets. The FGFR-tyrosine kinase inhibitor (TKI) pemigatinib has been introduced in the treatment of advanced cholangiocarcinoma and more recently for relapsed or refractory myeloid/lymphoid neoplasms with FGFR2 and FGFR1 rearrangements, respectively. Several clinical trials are currently investigating the possible combination of pemigatinib with immunotherapy.

In this study, we analyzed the biological and molecular effects of pemigatinib on different cancer cell models (lung, bladder, and gastric), which are currently objective of clinical trial investigations.

**Methods:**

NCI-H1581 lung, KATO III gastric and RT-112 bladder cancer cell lines were evaluated for FGFR expression by qRT-PCR and Western blot. Cell lines were treated with Pem and then characterized for cell proliferation, apoptosis, production of intracellular reactive oxygen species (ROS), and induction of senescence. The expression of microRNAs with tumor suppressor functions was analyzed by qRT-PCR, while modulation of the proteins coded by their target genes was evaluated by Western blot and mRNA. Descriptive statistics was used to analyze the various data and student’s t test to compare the analysis of two groups.

**Results:**

Pemigatinib exposure triggered distinct signaling pathways and reduced the proliferative ability of all cancer cells, inducing G1 phase cell cycle arrest and strong intracellular stress resulting in ROS production, senescence and apoptosis. Pemigatinib treatment also caused the upregulation of microRNAs (miR-133b, miR-139, miR-186, miR-195) with tumor suppressor functions, along with the downregulation of validated protein targets with oncogenic roles (c-Myc, c-MET, CDK6, EGFR).

**Conclusions:**

These results contribute to clarifying the biological effects and molecular mechanisms mediated by the anti-FGFR TKI pemigatinib in distinct tumor settings and support its exploitation for combined therapies.

**Supplementary Information:**

The online version contains supplementary material available at 10.1186/s12967-023-04450-7.

## Background

In the last decade, targeted therapies have revolutionized the treatment of cancer. Indeed, the possibility of selectively targeting cellular pathways essential to cancer growth and spreading by monoclonal antibodies and/or small molecules has shown a major impact on cancer patients’ outcomes and health [1].

Among them, tyrosine kinase inhibitors (TKIs) are small molecules developed to interfere with and inactivate the downstream kinase signaling of several receptors, such as vascular endothelial growth factor receptor (VEGFR), epidermal growth factor receptor (EGFR) and fibroblast growth factor receptor (FGFR) [2].

FGFRs and their ligands, the fibroblast growth factors (FGFs), are a large and complex family of molecules involved in a broad range of physiological mechanisms, related to development, proliferation, migration, and tissue homeostasis. Depending on the cell type, the binding of FGFs induces the dimerization and phosphorylation of FGFRs, activating downstream signaling through Ras/Raf-MEK-MAPKs, signal transducer and activator of transcription (STAT) and phosphatidylinositol 3-kinase PI3K/AKT [2]. Genetic changes, aberrant expression and altered FGF/FGFR signaling have been identified in several diseases [3]. In cancer, a dysregulated FGFR network is recurrent and associated with gene amplification, activating mutations, and oncogenic fusions [3]. FGFRs are aberrantly activated in cholangiocarcinoma (CCA) and most of the alterations involve the FGFR2 coding gene [4]. Upregulation of FGFR1 contributes to the progression and resistance in lung and breast cancer [5–8]. FGFR3 fusion with transforming acidic coiled-coil-containing protein 3 (TACC3) (FGFR-TACC3) has been characterized and correlated with a more aggressive phenotype in malignancies such as glioblastoma and head and neck cancers, for which no effective therapeutic option is still available [9]. The contribution of altered FGFRs to tumor development, progression and resistance to therapies indicates their importance as potential therapeutic targets [2]. Indeed, several TKIs blocking the FGF/FGFR axis have been developed and several of them are currently under investigation in phase I and II clinical trials in different tumor histotypes [10]. FGFR pathways are also mechanisms that mediate acquired resistance to other TKI treatments, thus FGFR blockade has been proposed to overcome acquired resistance to other TKI treatments, such as anti-EGFR and anti-c-Met [11, 12].

Pemigatinib (Pemazyre^®^, INCB054828) is a potent TKI targeting FGFR1-3 and rearranged forms, such as FGFR3-TACC3 and FGFR2-TRA2B, and its antitumor activity has been demonstrated in genetically defined tumor models [13]. Its clinical efficacy has been shown in patients harboring FGFR mutations [14–16]. In 2020, pemigatinib was approved by the FDA as the first targeted therapy in advanced cholangiocarcinoma and in August 2022 as a treatment for relapsed or refractory myeloid/lymphoid neoplasms (MLNs) with FGFR1 rearrangement [17, 18]. To date, several ongoing clinical trials are assessing the efficacy of pemigatinib, either alone or in combination with other therapeutic agents, in various cancers. These trials include patients who have not responded to standard therapies or have FGFR mutations, encompassing non-small cell lung cancer (NSCLC), bladder cancer, gastric cancer, colorectal cancer, and gliomas (NCT05210946, NCT05287386, NCT05253807; NCT03914794; NCT05559775; NCT05529667, NCT05202236; NCT04096417; NCT05267106; NCT04463771; NCT03914794) [10, 19]. Additionally, there is a terminated trial, NCT03822117, targeting agnostic tumors. Despite its expanding application in diverse clinical settings, little is known about the molecular and metabolic changes induced by pemigatinib. Elucidating these cellular mechanisms may contribute to fully explore the clinical potential of pemigatinib in different clinical settings.

Here we aim to investigate the efficacy of pemigatinib on cancer cell models, characterizing the biological effects and elucidating the molecular mechanisms that are triggered by pemigatinib treatment.

## Methods

### Cell lines

NCI-H1581 (H1581, ATCC CRL-5878) lung cancer and KATO III (ATCC HTB-103) gastric cancer cell lines were purchased from the American Type Culture Collection (ATCC; Washington, DC, NW) and cultured according to manufacturers’ instruction in Roswell Park Memorial Institute Medium (RPMI)—1640 with 5% heat-inactivated fetal calf serum (FCS; Merck KGaA, Darmstadt, Germany) and in Iscove’s modified Dulbecco’s medium (IMDM) with 20% FCS. The RT-112 (ACC 418) bladder cancer cell line was purchased from Leibniz Institute DSMZ- German Collection of Microorganisms and Cell Culture Gmbh and cultured according to the manufacturer’s instructions in RPMI with 10% FCS. Each cell line was maintained at 37 °C in 5% CO_2_.

### Reagents

Pemigatinib (Pemazyre^®^, INCB054828, Incyte Corporation) was dissolved in DMSO (Sigma‒Aldrich), stored at − 20 °C and used at different concentration (3 nM–1 μM).

### Cell lysate and Western blot

Cell lines were lysed using radioimmunoprecipitation assay (RIPA) buffer (1X, 100 ml/1 × 10^6^ cells, Cell Signaling, Beverly, MA, USA) with protease and phosphatase inhibitors (1X, Sigma‒Aldrich) for 30 min in ice and then centrifuged at 13,000 ×*g* for 10 min. Samples were aliquoted and stored at − 80 °C for further experiments. Protein content was quantified by Bradford assay using bovine serum albumin (BSA) as standard (Bio-Rad Lab, CA, USA).

Equal amounts of cell lysates were resuspended in sample buffer (Thermo Fisher Scientific, CA, USA), resolved using 4–12% NuPAGE™, Bis–Tris, 1.0–1.5 mm, Mini Protein Gels (NP0321BOX, Thermo Fisher Scientific) and transferred to nitrocellulose membranes. After blocking with Tris buffered saline with Tween 20 (T-BST) and 5% skim milk (SERVA Electrophoresis Gmbh, Heidelberg, Germany), membranes were incubated with the following antibodies at a concentration of 1:1000 rabbit anti-β-tubulin, anti- β-actin, anti-EGFR, anti-Met, anti-FGFR1, anti-FGFR2, anti- c-RAF and anti-phospho c-Raf, anti-AKT and anti-phospho AKT and mouse anti-GAPDH, anti-phospho p44/42 MAPK (Erk1/2) and anti-Phospho-Histone H2A.X (γ-H2A.X) all from Cell Signaling and anti-p21 (Abcam); 1:200 mouse anti-FGFR3 (Santa Cruz Biotechnology) and rabbit anti-ERK1/2 (Santa Cruz Biotechnology); 1:500 mouse anti-CDK6 (Santa Cruz Biotechnology), rabbit anti-c-Myc (Cell Signaling) and goat anti-TACC3 (R&D Systems); 1:4000 rabbit anti-lamin B (Abcam). Membranes were washed and incubated with peroxidase-conjugated goat anti-rabbit immunoglobulin G (IgG, H + L) (1:20,000), peroxidase-conjugated goat anti-mouse IgG (H + L) (1:20,000), and peroxidase-conjugated donkey anti-goat IgG (H + L) (1:20,000), all from Jackson ImmunoResearch Laboratories, West Grove, PA USA. Protein bands were detected with horseradish peroxidase (HRP)-enhanced chemiluminescence (ECL) (Advansta, CA, USA), following the manufacturer’s instructions. The density of protein bands was analyzed by ImageJ software and was normalized in terms of the average intensity of bands of each protein per the average intensity of bands of β-tubulin, β-actin or GAPDH. Data were obtained as mean ± SEM of three independent experiments.

### RNA extraction and RT-qPCR

RNA from cell lines was obtained by an automated Maxwell RSC-Promega extractor using the Maxwell RSC miRNA Tissue Kit (CAT # AS1460, Promega). For microRNA (miRNA) expression analysis, a miRNA-specific RT was carried out using Taq-Man™ MicroRNA Reverse Transcription Kit, while The High-Capacity cDNA reverse transcription kit (Applied Biosystems Life Technologies, ThermoFisher) was used to synthesize cDNA for mRNA expression. TaqMan Individual microRNA assays (Cat. N.4427975, Thermo Fisher Scientific) were used to assess the expression of hsa-miR-133b (Assay ID: 002247), hsa-miR-139 (Assay ID: 002289), hsa-miR-186 (Assay ID: 002285), hsa-miR-195 (Assay ID: 000494) and U6 snRNA (Assay ID: 001973). U6 was used as a normalization control.

Primers for mRNA expression were as follows: c-MYC (Assay ID: Hs00153408_m1), EGFR (forward 5′-GGCCGACAGCTATGAGATGG-3′; reverse: 5′- TTCCGTTACACACTTTGCGG -3′), MET (forward: 5′-CTGCCTGCAATCTACAAGGT-3′; reverse: 5′-ATGGTCAGCCTTGTCCCTC-3′), CDK6 (forward: 5′-GCTCTAACCTCAGTGGTCGT-3′; reverse: 5′-TGGACTGGAGCAAGACTTCG-3′), β-ACTIN (forward: 5′-ATGGAAGAAGAGATCGCCGC; reverse: 5′-TCGTAGATGGGCACCGTGTG-3′). qPCR was performed using Applied Biosystems ViiA 7 Real-Time PCR (Thermo Fisher Scientific, Waltham, MA, USA). The relative expression levels of miRNAs and mRNAs were calculated and quantified using the 2^-∆∆Ct^ method. All procedures were performed according to the manufacturer’s instructions and in three independent experiments. Data were reported as mean ± SEM of the three independent experiments.

### MTT assay

Cancer cells were seeded in 96-well plates (Corning Incorporated, New York, USA) (H1581 8 × 10^4^ cell/mL; KATO III 6 × 10^4^ cell/mL; RT-112 4 × 10^4^ cell/mL) and allowed to stabilize overnight. Cells were then treated with serial dilutions of pemigatinib (3–1000 nM; each condition in triplicate). Untreated cells (NT) were used as the experimental control. At the end of 24 h of incubation, MTT assays (Roche Diagnostics, Basel, Switzerland) were performed according to the manufacturer’s instructions. Absorbance was measured at 550 nm. Data were reported as mean ± SEM of three independent experiments.

### Apoptosis assay and cell cycle assay

Cancer cell lines were seeded in T25 flasks (Falcon, 353109) according to the manufacture’s instruction, allowed to stabilize overnight, and then treated with pemigatinib (100 nM) for 24 h and 48 h. Untreated cells (NT) were used as controls. At the end of treatment, KATO III and RT-112 cells were harvested by trypsinization (1x, Sigma‒Aldrich) and H1581 cells were resuspended in PBS and washed. After washing for 1200 rpm × 5 min, cells were used for apoptosis and cell cycle assays. For the apoptosis assay, cells were resuspended at 10^6^ cells/mL in 1 × Annexin V Binding Buffer (BD Biosciences). Cells were stained with 7-AAD and Annexin V-FITC (BD Biosciences) for 15 min.

For the cell cycle assay, the cells were fixed in 70% cold ethanol, at 4 °C overnight. The cells were incubated with RNaseA (Sigma‒Aldrich) for 30 min at RT and then incubated with propidium iodide (PI, BD Pharmigen, San Diego, CA, USA). Both analyses were performed using FACSCanto II flow cytometer as previously described and data were reported as mean ± SEM of three independent experiments.

### Calreticulin membrane exposure evaluation

Cell lines were seeded in 6-well plates and allowed to adapt overnight in culture. The cells were treated with pemigatinib (100 nM) for 24 h and 48 h. Untreated cells (NT) were used as experimental controls. Calreticulin (CRT) membrane exposure was evaluated by flow cytometry, using an anti-calreticulin mouse primary antibody (Abcam, 1:100). Then, the cells were washed (2x), and incubated with PE-conjugated anti-mouse IgG (Southern Biotech Limited, USA). Flow cytometry was performed as previously described. Data were derived from three independent experiments.

### ROS production

Cells were seeded in 6-well plates (H1581: 1 × 10^5^ cells/mL; KATO III: 1 × 10^5^ cells/mL; RT-112: 6 × 10^4^ cells/mL) and allowed to adapt overnight. The cells were incubated with pemigatinib (100 nM) for 48 h. Untreated cells (NT) were used as experimental controls. Cells treated with H_2_O_2_ for 1 h in 37 °C 5% CO_2_ incubator were used as positive control. After treatment, cells were washed with PBS, and the adherent cells (KATO III and RT-112) were immediately incubated with 10 mM DCFDA/H2DCFDA (Abcam, ab113851) for 15 min in an incubator. Then, the cells were washed and harvested with trypsin. H1581 cells were washed and then incubated with 10 mM DCFDA/H2DCFDA (Abcam) in a 5 mL FACStube (Falcon, 352054) and then washed. The analysis was performed using FACSCanto II and FlowJo Software (BD, Biosciences). For the immunofluorescence assay, the cells were seeded on a coverslip and after probe incubation were analyzed, employing Apotome Microscope (40X magnification). The results were derived from three independent experiments.

### β-Galactosidase staining

Cells were seeded in 6-well plates (H1581: 1 × 10^5^ cells/mL; KATO III: 1 × 10^5^ cells/mL; RT-112: 6 × 10^4^ cells/mL) and allowed to adapt overnight. Cells were incubated with pemigatinib (100 nM) for 48 h. Untreated cells (NT) were used as the experimental control. The Senescence β-Galactosidase Staining Kit (Cell Signaling Technology, Hitchin, Herts, UK Cat no. 9860) was used to measure senescence according to the manufacturer’s instructions. β-Galactosidase Staining was performed in three independent experiments.

### Ki67 analysis

Cells were seeded in 6-well plates (H1581: 1 × 10^5^ cells/mL; KATO III: 1 × 10^5^ cells/mL; RT-112: 6 × 10^4^ cells/mL) and allowed to adapt overnight. Cells were incubated with pemigatinib (100 nM) for 24 h and 48 h. Untreated cells (NT) were used as the experimental control. Ki67 intracellular concentration was analyzed by flow cytometry, using an anti-human-Ki67-BV421 (BD, Bioscience, 1:200). Flow cytometry was performed as previously described and data were reported as the means of three independent experiments.

### ATP and HMGB1 release

Cell lines were seeded in 6-well plates and allowed to adapt overnight in culture. The cells were treated with pemigatinib (100 nM) for 24 h and 48 h. Untreated cells (NT) were used as experimental controls. Culture supernatants were collected by centrifugation and immediately used for ATP and HMGB1 release assays. ATP release in the supernatant of cells was measured by means of an ENLITEN ATP Assay kit (Promega, Fitchburg, WI, USA), based on the ATP-dependent luciferin conversion, which yields detectable bioluminescence, according to the manufacturer’s protocol. HMGB1 concentrations in the supernatant of cells were measured by means of an enzyme-linked immunosorbent assay (ELISA) kit (TECAN, Zürich, Switzerland), according to the manufacturer’s protocol. All experiments were conducted in triplicates.

### Statistical analysis

Statistical analysis was performed using GraphPad Prism version 8 (Graphpad Software, Inc., San Diego, USA). Descriptive statistics [average and standard error media (SEM)] was used to describe the various data. Student’s paired t-test was used to compare two groups. Fold change represents the ratio between values obtained at treated and not treated cells (T/NT). The results with a* p* value < 0.05 were considered statistically significant *p < 0,05; **p < 0,01; ***p ≤ 0,001; ****p ≤ 0,0001.

## Results

### FGFR targeting by pemigatinib impairs cancer cell growth by arresting the cell cycle in G1 phase

The expression of FGFRs targeted by pemigatinib (Pem) was investigated in several cancer cell lines by qRT‒PCR and Western blot, and the H1581 lung, KATO III gastric and RT-112 bladder cancer cell lines were selected for their distinct expression of FGFRs (Additional file 1: Table S1).

**Fig. 1:**
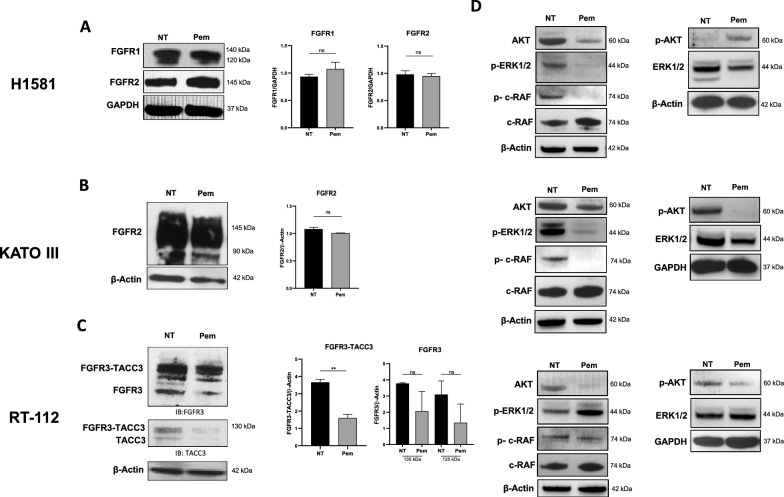
FGFR expression profile in cancer cell lines and downstream signaling pathways. **A** Expression of FGFR1 and 2 in H1581 lung cancer cells by Western blot. GAPDH was employed as reference marker for relative protein expression and histograms represent the relative band intensity calculated as mean ± SEM of three independent experiments. **B** Expression of FGFR2 in KATO III gastric cancer cells by Western blot. β-actin was employed as reference marker for relative protein expression and histograms represents the relative band intensity calculated as mean ± SEM of three independent experiments. **C** Expression of FGFR3 and FGFR3-TACC3 fusion in RT-112 bladder cancer cell line by Western blot analysis. The FGFR3 western blot (left panel) recognizes both native FGFR3 and the FGFR3-TACC3 fusion protein with slightly higher molecular weight. The TACC3 western blot (panel below) recognizes both native TACC3 and the FGFR3-TACC3 fusion protein with slightly higher molecular weight. β-actin was employed as reference marker for relative protein expression and histograms represent the relative band intensity calculated as mean ± SEM of three independent experiments. **D** Western blot analysis of c-RAF/p–c-RAF, AKT/p-AKT, ERK1/2/p-ERK1/2 in untreated (NT) and treated (Pem) H1581 (upper panels), KATO III (middle panels) and RT-112 (bottom panels) cells. GAPDH and β-actin were employed as reference markers. **p < 0.01; ns, not significative

As shown in Fig. 1, H1581 cells expressed FGFR1 and FGFR2, while KATO III cells overexpressed FGFR2. RT-112 cells did express both FGFR3 and the FGFR3-TACC3 fusion protein. To identify the optimal Pem concentration for the treatment in the selected cancer cell lines, serial dilutions of Pem (3 nM–1 μM) were used and cell proliferation was evaluated at 24 and 48 h employing MTT assay and analyzing the expression of the nuclear Ki67 factor, a well-known marker of cell proliferation (Additional file 1: Fig. S1A, B).

The minimum concentration of Pem to which we observed the optimal proliferation decrease for most of the cells was 100 nM in both assays. This selected concentration corresponded to the maximum plasmatic concentration of Pem found in the blood of cancer patients after drug administration [20, 21].

Pem treatment did not alter the expression levels of FGFR1 and FGFR2 in H1581 and KATO III cells (Fig. 1A, B), while it induced a trend toward a reduction in FGFR3 expression levels and a significant reduction in the FGFR3-TACC3 fusion protein (*p* = 0.0087) in RT-112 cells (Fig. 1C). We analyzed the downstream signaling [22] as shown in Fig. 1D (upper panels), Pem induced a reduction of AKT, p–c-RAF, and both native and phosphorylated ERK1/2, and a slight upregulation of p-AKT and c-Raf in H1581 cells. Treated KATO III cells displayed a clear downregulation of all the phosphorylated kinase forms i.e., p-ERK1/2, p–c-RAF, and p-AKT, and a mild downregulation of ERK1/2 suggesting that Pem could impair both PI3K/AKT and ERK1/2 signaling pathways in gastric cancer cell line (Fig. 1D, middle panel). RT-112 showed a reduction of both total and p-AKT and total ERK1/2 and an upregulation of p-ERK1/2 (Fig. 1D, bottom panels).

**Fig. 2:**
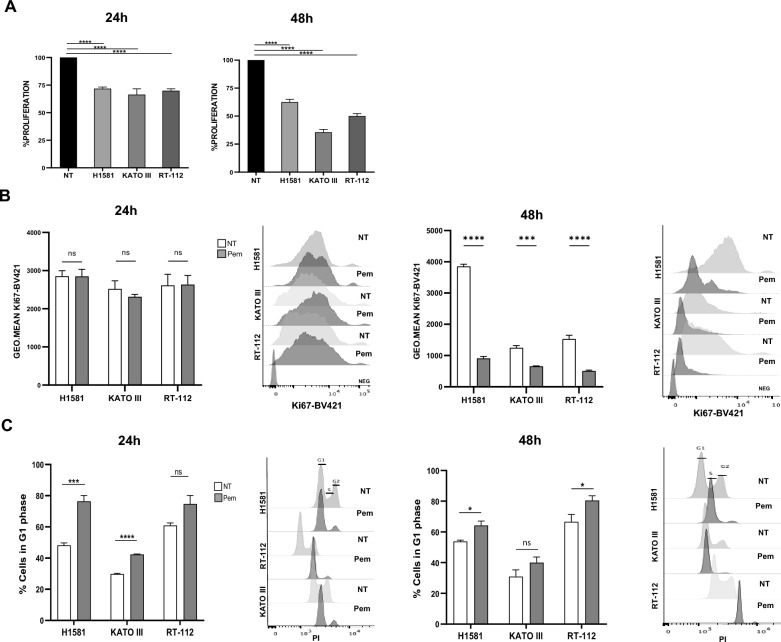
Pemigatinib treatment modulates proliferation and cell cycle arrest in cancer cell lines. **A** Effect of Pem (100 nM) on the proliferation of H1581, KATO III and RT-112 cells after 24 h and 48 h; **B** The flow cytometry histogram plots and bar-plots represent the mean ± SEM of Ki67-BV421 geometric mean of fluorescence in untreated (NT) and Pem-treated (100 nM) H1581, KATO III and RT-112 cells. **C** Effect of Pem (100 nM) on the G1 phase-cell cycle. Cell cycle analysis was performed by propidium iodide (PI) staining by flow cytometry. The flow cytometry histogram plots are representative of the cell cycle of one experiment at 24 h and 48 h and the histogram bar-plots represent the count of PI-positive cells found in G1 phase in untreated (NT) and Pem-treated (100 nM) H1581, KATO III and RT-112 cells; All experiments are represented as the mean of three independent experiments ± SEM. *p < 0.05; **p < 0.01; ***p ≤ 0.001; ****p ≤ 0.0001. Student’s *t* test

To test the effect of Pem on cell viability and proliferation, we exposed H1581, KATO III, and RT-112 cells to the drug (100 nM) for 24 and 48 h. Untreated cancer cells (NT) were used as experimental controls. Pem significantly affected the viability of each cancer cell line at both time points (*p* < 0.0001), resulting in a reduction of 25% after 24 h and 40–60% after 48 h of treatment, as detected by MTT assays (Fig. 2A). A marked decrease in cell proliferation was observed after 48 h in all cancer cell lines (*p* < 0.001, Fig. 2B), measured as Ki67 levels. Cell cycle perturbation might make account for the proliferative decrease observed. Indeed, a cell cycle block was observed following Pem treatment. As shown in Fig. 2C, Pem induced the arrest of H1581 and KATO III cancer cells in the G1 phase after 24 h (*p* = 0.0004 and *p* < 0.0001, respectively). This block was maintained at 48 h in H1581 cells (*p* = 0.014) but not in KATO III cells. RT-112 cells showed a trend in G1 phase arrest at 24 h (*p* = 0.07), that became significant after 48 h of treatment (*p* = 0.049). Accordingly, a reduction in the S phase was observed at both time points for all cancer cell lines following Pem treatment. The G2 phase was also reduced in the H1581 and RT-112 cell lines, while no significant change was observed in the KATO III cells (Additional file 1: Fig. S2).

Taken together, these results suggest that Pem exerted a cytostatic effect on cancer cells by blocking the cell cycle in the G1 phase and therefore reducing cell proliferation.

### Pemigatinib triggers cellular stress inducing apoptosis, senescence, ROS production and calreticulin expression

The cell cycle alteration induced by Pem could underline a strong cellular stress condition and several cellular mechanisms may occur.

**Fig. 3:**
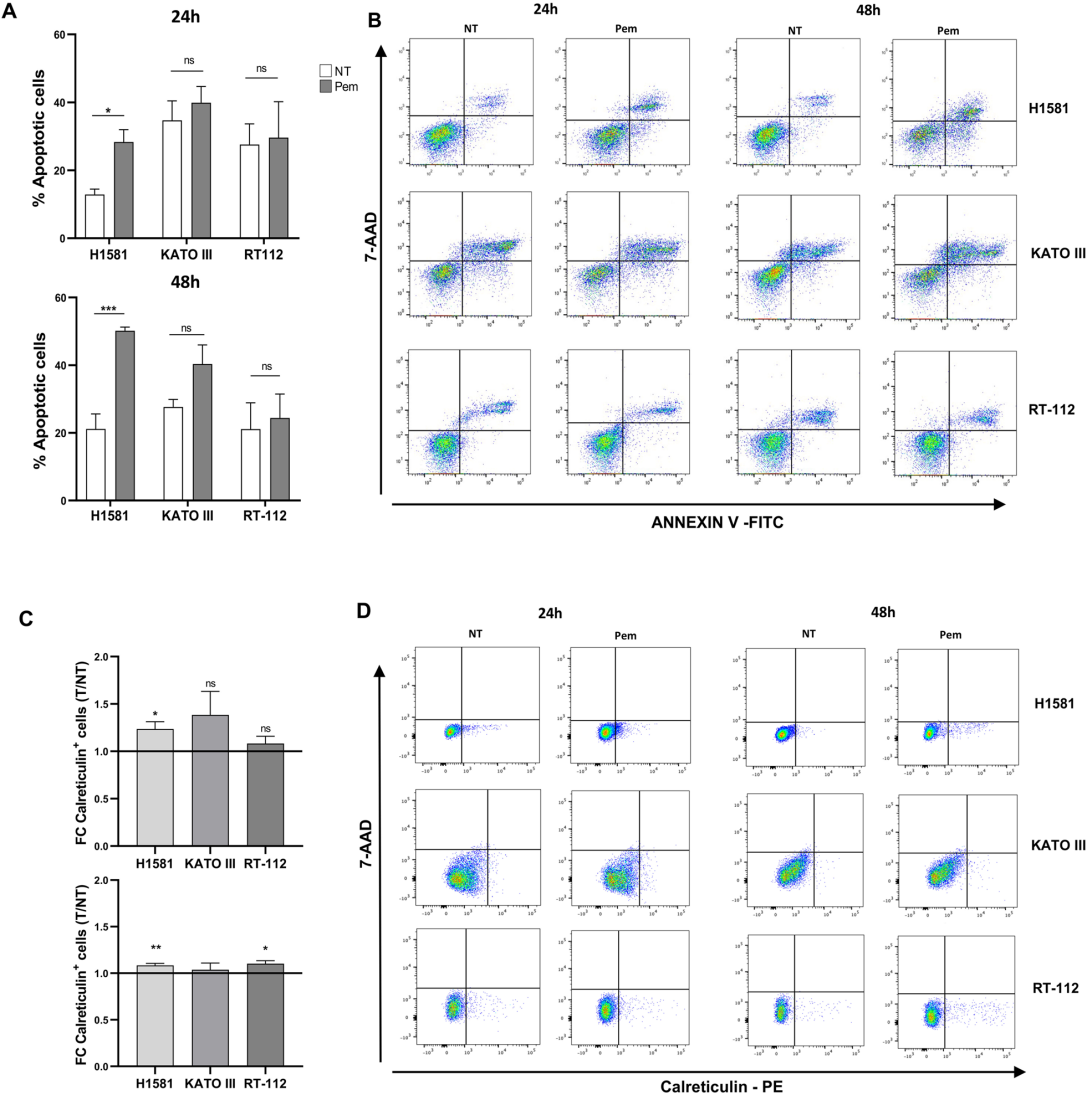
Effect of pemigatinib on apoptosis and calreticulin exposure in cancer cell lines. **A** Apoptosis of cancer cells exposed to Pem (100 nM) at 24 h and 48 h. Untreated cells (NT) were employed as control. Apoptosis was detected as Annexin V-7-AAD staining by flow cytometry. Histograms correspond to the average percentage of apoptotic cells of three independent experiments ± SEM. *p < 0.05; **p < 0.01; ***p ≤ 0.001. **B** Dot-plot graphs are representative of the expression of Annexin V and 7-AAD nuclear intercalant of one experiment for each cancer cell line untreated (NT) and treated with Pem. **C** Calreticulin cell surface expression after Pem exposure (100 nM) was evaluated by flow cytometry. The results are plotted as the ratio between the percentages of calreticulin-positive pemigatinib-treated cells and calreticulin-positive Pem-untreated cells. **D** Dot-plot graphs are representative of the expression of Calreticulin-PE and 7-AAD nuclear intercalant of vital cells of one experiment for each cancer cell line untreated (NT) and treated with Pem. Each histogram represents the average of values of 3 independent experiments. Variability is expressed as the SEM. *p < 0.05; **p < 0.01; ***p ≤ 0.001

Apoptosis induction was first analyzed. Apoptosis was observed only in H1581 cells at 24 h of treatment (*p* < 0.018), becoming more pronounced after 48 h (*p* = 0.0007*).* This phenomenon was not observed for KATO III and RT-112 cells after Pem treatment*,* although KATO III showed a trend toward an increase in apoptosis after 48 h of exposure to the drug *(p* = 0.08) (Fig. 3A, B).

Translocation of ER-resident proteins on the plasma membrane surface is another sign of cellular distress, and the expression of surface calreticulin (CRT) was analyzed. H1581 cells showed a significant increase of CRT at both time points* (*24 h*, p* = 0.020; 48 h, *p* = 0.009*),* while RT-112 cells showed a significant increase only at 48 h (*p* = 0.017). No changes in CRT levels were observed in KATO III cells (Fig. 3C, D).

**Fig. 4:**
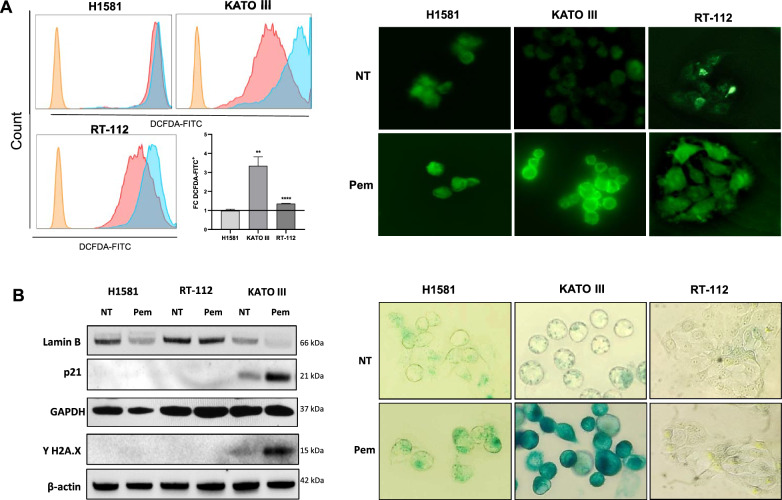
Effect of pemigatinib on ROS and senescence in cancer cell lines. **A** Flow cytometry plots (left panel) and immunofluorescence staining (40 × magnification) with DCFH-DA probe (right panel) of intracellular ROS in cancer cells untreated and treated with Pem (100 nM). The histogram results are plotted as the fold change in the MFI of the treated (light blue) *vs.* the untreated samples (pink) of three independent experiments. *p < 0.05; **p < 0.01; ***p ≤ 0.001. **B** Cellular senescence. Western blot of lamin B, p21 and γ-H2A.X protein expression in untreated (NT) and treated (Pem) H1581, RT-112 and KATO III cells at 48 h. GAPDH and β-actin were used as reference markers (left panel). β-Galactosidase staining of untreated and Pem-treated H1581, KATO III and RT-112 cells at 48 h was assessed with a magnification of 40X under a Leyca microscope (right panel). The β-Galactosidase staining assay was performed in three independent experiments

Frequently, cell proliferation arrest triggers an increase in reactive oxygen species (ROS) production. As shown in Fig. 4A, KATO III and RT-112 cells notably increased intracellular ROS levels (*p* = 0.008 and *p* < 0.0001) upon Pem treatment as analyzed by flow cytometry. H1581 cells already exhibited maximum levels of intracellular ROS at baseline, that were not augmented following Pem exposure. Similar results were also obtained in the immunofluorescence assay.

Cell senescence is another irreversible mechanism characterized by the cessation of cell proliferation without undergoing cell death and by specific biochemical changes such as p21 and lamin B modulation [23]. As shown in Fig. 4B, Pem treatment increased p21 and decreased lamin B levels in KATO III cells, while no change was observed in H1581 and RT-112 cells. Senescence is often associated with DNA damage; therefore, all cancer cells were also evaluated for the expression of γ-H2A.X, a DNA damage marker. As expected, we observed an increased expression of γ-H2A.X only in KATO III cells, upon Pem treatment (Fig. 4B). This marker was not upregulated in the other cancer cells. These results were also confirmed by β-galactosidases assay: after 48 h Pem treatment, KATO III cells showed marked staining, accompanied by mild morphological changes while no change was observed in the H1581 and RT-112 cells (Fig. 4B).

These results suggest that Pem could induce several metabolic pathways related to cellular stress in distinct cancer cells and that these different effects could depend on the distinct FGFR target expressed and the intrinsic tumor features.

### Pemigatinib treatment increases tumor suppressor miRNAs

MicroRNAs (miRNAs) are master regulators of gene expression and have a critical role in the control of several aspects of cell biology, including the cell cycle, apoptosis and cell metabolism [24]. Target therapies can modulate miRNA production, so affecting the response to therapy [25, 26]. To investigate whether the effect of Pem on cancer cell features involves modulation of miRNAs and their targets, we selected miRNAs with known tumor suppressor roles implicated in the tumorigenic processes of lung, gastric and bladder cancers [27–29].

**Fig. 5:**
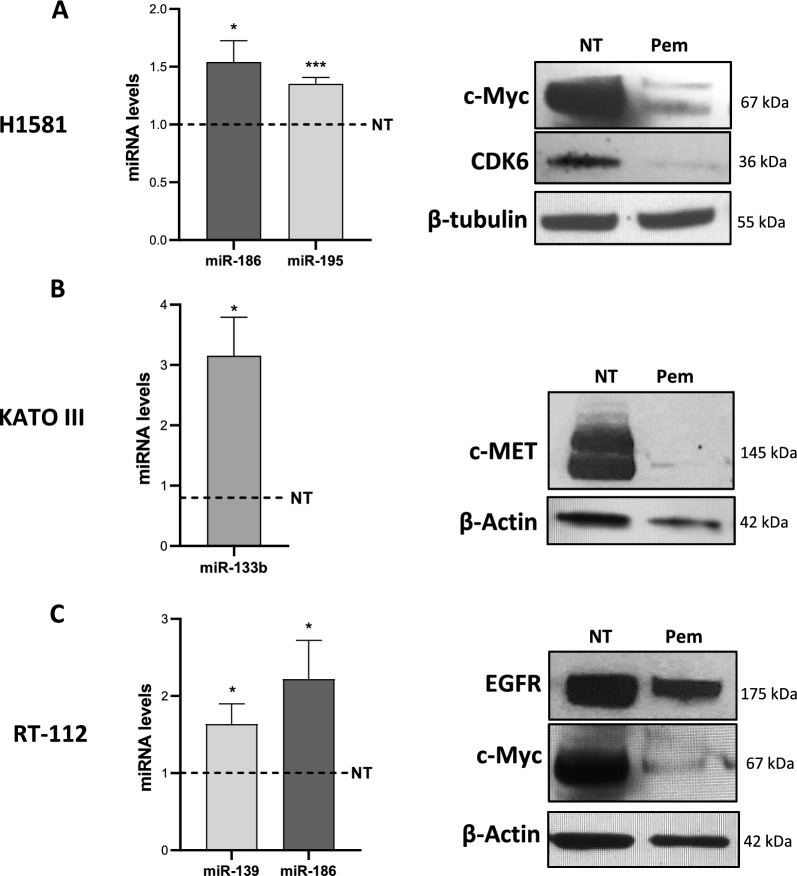
Expression levels of selected miRNA and protein targets in cancer cell upon pemigatinib treatment. **A** Left: histograms show the expression of miR-186 and miR-195 and right: Western blots of CDK6, target of miR-195, and c-Myc, target of miR-186 and miR-195 in H1581 cells treated with Pem for 48 h. **B** Left: histogram showing the expression of miR-133b and right: Western blots of MET, a target of miR-133b, in KATO III cells treated with Pem for 48 h. **C** Left: histogram showing the expression of miR-139 and miR-186 and right: Western blots of EGFR, target of miR-139, and c-Myc, target of miR-186, in RT-112 cells treated with Pem for 48 h. Histograms represent the mean ± SEM of three experiments, and the dashed line indicates the control (NT). *p < 0.05; ***p ≤ 0.001; Student’s *t*-test

In particular, for the H1581 cells we selected miR-186 and miR-195 that have been shown to play a tumor suppressor function in NSCLC [30, 31]. miR-186 has also shown to regulate invasion and metastatic potential of bladder cancer cells [32] as well as miR-139 [33]: both miR-186 and miR-139 modulation was investigated in RT-112 cancer cells. miR-133b was analyzed in the KATO III since it has been found to attenuate tumorigenesis in gastric cancers [34]. Upon Pem treatment all selected miRNAs were significantly upregulated in the cancer cell lines after 48 h of treatment (Fig. 5). MiR-186 expression increased in H1581 and RT-112 cells upon Pem treatment (*p* = 0.02 and *p* = 0.04, respectively), while miR-195 (*p* = 0.001), miR-133b (*p* = 0.02) and miR-139 (*p* = 0.04) were upregulated in H1581, KATO III and RT-112 cells, respectively.

To better investigate the malignant pathways impacted by Pem treatment, we also selected specific miRNA-mRNA targets implicated in proliferative and cell cycle processes by interrogating the publicly available database miRTarBase [71]. We focused on cyclin-dependent kinase 6 (CDK6) as a target of miR-195, c-Myc as a target of miR-186, c-Met as a target of miR-133b and EGFR as a target of miR-139, and we checked the expression the protein products by Western blot in each corresponding cell lines. The results showed that all protein encoded by the miRNA targeted genes decreased after Pem treatment. Indeed, in H1581 cells, CDK6 and c-Myc proteins were downregulated (Fig. 5A), as well as c-MET in KATO III cell line (Fig. 5B). In RT-112 cells, c-Myc and EGFR proteins were downregulated in correspondence with increases in miR-139 and miR-186, respectively (Fig. 5C). Interestingly, mRNA levels of c-Myc were also downregulated in H1581 and RT-112 cells (Additional file 1: Fig. S4). Our results suggest that Pem can impact the mechanisms of proliferation and the cell cycle in cancer cells by modulating the expression of selected miRNAs.

## Discussion

The selective targeting of TK receptors that sustain tumor biology has shown promising results in restraining tumor progression and aggressiveness. Depending on the target, TKIs can affect tumor cell vitality, proliferation, migration and the surrounding microenvironment, such as blood vessel construction and immunity [35–37]. Increasing evidence has shown that targeted therapies can have an “off-target” effect on immune cells. Indeed, several TKIs have shown a strong ability to modulate immune cell subsets, thus promoting an antitumor immune response [38, 39].

Understanding the direct and indirect effects of targeted therapies is crucial to identify and develop therapeutic combinations/sequences that could harness the nonoverlapping mechanisms of action of the different therapeutic agents to achieve the most successful outcome for cancer patients.

In this work, we described the direct effects of pemigatinib on three different cancer cell lines expressing different FGFRs. Pemigatinib is a TKI targeting the FGFR pathway that was first approved by FDA for the treatment of cholangiocarcinoma [17, 40]. This inhibitor differs from earlier TKIs with FGFR‒targeted activity due to its high selectivity for FGFR1, FGFR2, and FGFR3, making this inhibitor particularly attractive for the treatment of FGFR-driven cancers.

Pemigatinib exerts its biological function by reducing tumor cell growth as demonstrated in vitro in prostate cancer cells and in vivo in mouse models [41]. Similar evidence was also obtained with other TKIs targeting FGFR, such as erdafitinib, which reduced the proliferation of lung cancer cells and decreased the level of c-Myc and its target genes [42]. In particular, S-phase cell cycle arrest seems to be induced by erdafitinib [43].

Our results demonstrated that FGFR blockade by pemigatinib results in a significant reduction in tumor proliferation by arresting the cell cycle at the G1 phase in all the employed cancer cell lines. These results suggest that the G1-phase arrest mediated by pemigatinib is a shared cytostatic mechanism among different FGFR expression patterns and tumor types. Cell cycle arrest usually underlines a cellular stress condition that could involve several metabolic pathways. In fact, in our tumor models, pemigatinib treatment differently triggered the downstream signaling pathways that we investigated i.e., PI3K/AKT and RAF/MEK/ERK. These signaling modules are involved in cell growth, proliferation, migration and metabolic function [44, 45].

Pemigatinib treatment downregulated the RAF/ERK pathway in both lung H1581 and gastric KATO III cells, that share the expression of FGFR2. However, KATO III also displayed a marked downregulation of the PI3K/AKT signaling. In the bladder RT-112 cells, the inhibition of FGFR3 and its chimera FGFR3-TACC3 led to the modulation of AKT signaling. These distinct signaling profiles are conceivable with the different FGFR expression and affinity for the target. Furthermore, the complex interrelation among the signaling transduction pathways may result in the upregulation of specific pathways as compensatory mechanisms as observed in RT-112 with the increase of p-ERK1/2, when p-AKT is completely downregulated. Indeed, the compensatory activation of PI3K/AKT and MEK/ERK signaling pathways have been proven and it has been associated with resistance mechanisms to pharmacological treatments in a wide range of human malignancies [46]. These effects increase the complexity of the signaling balance and may result in distinct and diverse metabolic processes as we observed in the three cell lines.

H1581 lung cancer cells underwent apoptosis after 24 and 48 h of pemigatinib treatment. This phenomenon was already observed in prostate cancer cell lines treated in vitro with pemigatinib [41] and in the H1581 lung cancer cell line after the administration of erdafitinib [42]. The apoptosis of lung cancer cells was also accompanied by increased expression of calreticulin (CRT), which was also observed in RT-112 bladder cells after 48 h of treatment. CRT overexpression was identified as a marker associated with strong cellular distress, which promotes the uptake of cell corpses by phagocytes that ultimately supports the initiation of antitumor immunity [47]. CRT membrane overexpression is also considered a hallmark of immunogenic cell death (ICD) when it occurs in combination with the extracellular release of adenosine triphosphate *(ATP)* and high mobility group box-1 (HMGB1) molecules [48]. Here, CRT overexpression was not accompanied by changes in HMGB1 and ATP (Additional file 1: Fig. S3), suggesting that pemigatinib did not trigger ICD, as observed for other TKIs directed against different molecular targets [49] although inducing a strong cellular stress. Indeed, our data also demonstrated that pemigatinib increased the levels of cytoplasmic ROS in RT-112 and KATO III cell lines. Lung H1581 cancer cells showed elevated baseline levels of intracellular ROS, which were not further increased by the addition of the drug. Similar results were observed in lung cancer and multiple myeloma cancer cell models in which pemigatinib increased the cytoplasmic and mitochondrial ROS accompanied by mitochondrial membrane depolarization [42, 50], thus confirming the hypothesis that the involvement of the FGF/FGFR pathway is crucial for the maintenance of metabolic and redox equilibrium.

Frequently, mitochondrial dysfunction with ROS production can occur in senescent cells [51]. In our tumor models, the gastric cancer cell line KATO III exhibited a high percentage of senescent cells associated with a high increase in cytoplasmic ROS after pemigatinib treatment. The reduction of lamin B expression concurrently with the upregulation of p21 in treated KATO III cells indicates the induction of senescence upon pemigatinib treatment. Interestingly, this phenomenon was also accompanied by the increase of γ-H2A.X expression, marker of DNA damage, which often is associated to senescence process [52].

Viability, cell growth and metabolic changes that occur upon pemigatinib treatment can also be mediated by the modulation of miRNA, which are important modulators of cell biology and are often altered in cancer development and progression. Indeed, an oncosuppressor role has been described for many miRNAs in the cancer contexts we investigated [27–29]. Specifically, miR-186 and miR-195 have been shown to inhibit proliferation, migration and invasion in NSCLC cancer cell lines and tissue biopsies [30, 31, 53–57]. Moreover, miR-186 serum levels were higher in NSCLC patients with higher grade tumors and metastasis than in stage I-II NSCLC patients. MiR-133b is usually downregulated in gastric cancer, and its dampening is associated with a more aggressive phenotype [34, 58–60]. In bladder cancer, both miR-186 and miR-139 have been described as relevant tumor suppressors and their downregulation is associated with invasion and metastasis [32, 33].

Our results show a positive modulation of tumor suppressor miRNAs in all tested cancer cell lines, underlining a possible contribution of miRNA machinery to pemigatinib-induced effects. To our knowledge this is the first report describing upregulation of tumor suppressor miRNA upon pemigatinib treatment as well as other FGFR inhibitors. The miRNA-targeted mRNAs we analyzed encode for proteins that are involved in cell cycle and proliferation. Their reduced expression following pemigatinib exposure was in accordance with the biological effects we observed. c-Myc is targeted by the oncosuppressors miR-186 and miR-195 and pemigatinib treatment reduced its expression in all the tested cell lines.

c-Myc is a master regulator of metabolic and biosynthetic transcriptional pathways driving cell growth and proliferation [61]. So, c-Myc downregulation correlated with the proliferative reduction we observed in the FGFR cell models, showing a G1 phase cell cycle arrest. Notably, erdafitinib, a FGFR TKI, has been shown to downregulate c-Myc protein expression [42]. These results likely confirm that c-Myc modulation is a common key-point of FGFR blockade, regardless of the tumor histotype. Interestingly, the downregulation of the c-Myc protein was also associated with a significant decrease of its coding mRNA. Usually, miRNAs act on gene expression at post-transcriptional level by translational repression, but in some cases this can be accompanied by mRNA decay [62]. Thus, the decrease of c-Myc transcript levels may be due to a combination of miRNA-mediated and miRNA-independent mechanisms. In addition, the reduced expression of CDK6, EGFR and c-Met triggered by pemigatinib is in accordance with the increased levels of miR-195, miR-139 and miR-133b, respectively.

In recent years, TKI-based therapies have been widely applied for the treatment of a large variety of tumor histotypes, conferring high clinical benefits and becoming standard therapies [35]. Unfortunately, the onset of acquired resistance is a common event and dramatically hampers therapeutic efficacy [63]. FGFR signaling pathways have been described as molecular mechanisms for acquired resistance to TKIs targeting other tyrosine kinase receptors, such as EGFR, c-Met, ALK and CDK4/6 [64–66]. Although functional studies are needed, the evidence that pemigatinib treatment can modulate other TKI targets reinforces the biological rationale for combinatorial strategies to overcome TKI resistance and improve clinical benefit.

It is interesting to note that CDK6 expression levels were reduced upon pemigatinib treatment. Indeed, FGFRs contribute to cyclin-dependent kinase 4/6 inhibitor (CDK4/6i) resistance and FGFR1 overexpression reduced the efficacy of CDK4/6i treatment for breast cancer patients enrolled in the MONALEESA-2 study [67].

CDK6 is a proliferative checkpoint pathway that is not exclusive to tumor cells but is also employed by immune cells. Targeting of this pathway by CDK4/6i has been shown to exert off-target immunological effects described in mouse models and is associated with response in cancer patients [68, 69]. Recent evidence indicates that FGFR blockade may modulate immune cell recruitment and infiltration at the tumor bed by remodeling the tumor microenvironment as well as modulating immune checkpoint molecule expression [70].

Although further studies are required to better investigate the possible off-target effects of pemigatinib on the immune compartment, the results until now available are supportive of the combination of pemigatinib with immunotherapy regimens and phase I clinical studies are already ongoing (NCT04949191, NCT05004974).

## Conclusions

In conclusion, pemigatinib exerted a cytostatic effect with G1 phase arrest shared by the cell models of different histotypes with distinct FGFR expression profiles.

The proliferative arrest induced stress signals e.g., intracellular ROS production and calreticulin membrane surface exposure. Additionally, apoptosis and senescence were induced by pemigatinib treatment. The transcription levels of miRNAs with oncosuppressor activity were increased (miR-186, miR-195, miR-139, miR-133b), concurrently the target proteins were downregulated (c-Myc, CDK6, EGFR, c-Met) suggesting that pemigatinib antitumor activity is at least in part mediated by miRNA modulation. These distinct effects may be related to the specific modulation of the FGFR downstream signaling pathways i.e., PI3K/AKT and RAF/MEK/ERK that we observed in each cell line upon pemigatinib treatment.

Our results contribute to clarifying the biological effects and molecular mechanisms mediated by the anti-FGFR TKI pemigatinib on cancer cells, for its exploitation in distinct tumor settings and in combination therapies with other TKIs and immunotherapy.

### Supplementary Information

**Additional file 1: Table S1.** FGFR expression in cancer cell lines. FGFR expression was evaluated by Western blot and q-RT‒PCR experiments in H1581, KATO III, RT-112, RCC4plusVHL and DU-145 cancer cell lines employed as cell models of lung, gastric, bladder, renal and prostate cancer, respectively. **Figure S1.** Serial dilution of Pem and proliferation analysis. **A** For selection of the optimal drug concentration, H1581, KATO III and RT-112 cells were exposed to serial dilutions of Pem (3–1000 nM) for 24 h and 48 h, and MTT proliferation assay was then performed. Data are reported as the mean of three independent experiments ± SEM as variability **B** H1581, KATO III and RT-112 cells were exposed to serial dilutions of Pem (25–400 nM) for 24 h and 48 h, and intracellular Ki67 levels were then evaluated by flow cytometry. The histograms represent the geo-mean of Ki67-BV421 MFI found in untreated (NT) and Pem-treated H1581, KATO III and RT-112 cells and are represented as mean of three independent experiments ± SEM. *p < 0.05; **p < 0.01; ***p ≤ 0.001 Student’s *t* test. **Figure S2.** Effect of Pem on the S and G2 phases of cell cycle. The histograms represent the count of PI-positive cells found in S- and G2-phase in untreated (NT, white) and Pem-treated (100 nM, grey) H1581, KATO III and RT-112 cells and are represented as mean of three independent experiments ± SEM. *p < 0.05; **, p < 0.01; ***p ≤ 0.001. Student’s *t* test. **Figure S3.** Effect of pemigatinib on ATP and HMGB1 release. **A** Extracellular HMGB1 release upon Pem treatment. The extracellular HMGB1 release was measured by means of an enzyme-linked immunosorbent assay (ELISA) kit (TECAN, Zürich, Switzerland), according to the manufacturer’s protocol. Histograms represent the mean values of three independent experiments ± SEM of HMGB1 released by untreated cells (NT, white) and Pem-treated cells (grey) at 24 and 48 h. ns, not significative. **B** Extracellular ATP release upon Pem treatment. The ATP release was measured by ENLITEN-Promega KIT as luminescence signals. The histograms represent the mean value of ATP moles of three independent experiments ± SEM by untreated cells (NT, white) and Pem-treated cells (Pem, grey) at 24 and 48 h. ns, not significative. **Figure S4.** Effect of pemigatinib on miRNA target transcripts. mRNA expression of miRNAs targets in untreated (NT) and Pem-treated (100 nM) cancer cell lines. Histograms represent the mean values of three independent experiments ± SEM.

## Data Availability

The data that support the findings of this study are available from Incyte Biosciences International S.a.r.l., but restrictions apply to the availability of these data, which were used under license for the current study, and so are not publicly available. Data are however available from the authors upon reasonable request and with permission of Incyte Biosciences International S.a.r.l.

## Abbreviations

FGFR: Fibroblast growth factor receptor
TKI: Tyrosine kinase inhibitor
VEGF: Vascular endothelial growth factor receptor
EGFR: Epidermal growth factor receptor
FGFs: Fibroblast growth factors
STAT: Signal transducer and activator of transcription
CCA: Cholangiocarcinoma
TACC3: Transforming acidic coiled-coil-containing protein 3
MLNs: Myeloid/lymphoid neoplasms
NSCLC: Non-small cell lung cancer
ATCC: American Type Culture Collection
RPMI: Roswell Park Memorial Institute Medium
FCS: Fetal calf serum
IMDM: Iscove’s modified Dulbecco’s medium
RIPA: Radioimmunoprecipitation assay
BSA: Bovine serum albumin
T-BST: Tris buffered saline with Tween 20
NT: Untreated cells
CRT: Calreticulin
Pem: Pemigatinib
ROS: Reactive oxygen species
miRNAs: MicroRNAs
CDK6: Cyclin-dependent kinase 6
ICD: Immunogenic cell death
ATP: Adenosine triphosphate
HMGB1: High mobility group box-1
CDK4/6i: Cyclin-dependent kinase 4/6 inhibitor
