# Supplementary material for: Targeting FGFRs by pemigatinib induces G1 phase cell cycle arrest, cellular stress and upregulation of tumor suppressor microRNAs
Source: J Transl Med. 2023 Sep 15;21:626. doi: 10.1186/s12967-023-04450-7 (PMC10504800; doi:10.1186/s12967-023-04450-7)
Supplement: Supplementary file 1 — Additional file 1: Table S1. FGFR expression in cancer cell lines. FGFR expression was evaluated by Western blot and q-RT‒PCR experiments in H1581, KATO III, RT-112, RCC4plusVHL and DU-145 cancer cell lines employed as cell models of lung, gastric, bladder, renal and prostate cancer, respectively. Figure S1. Serial dilution of Pem and proliferation analysis. A For selection of the optimal drug concentration, H1581, KATO III and RT-112 cells were exposed to serial dilutions of Pem (3–1000 nM) for 24 h and 48 h, and MTT proliferation assay was then performed. Data are reported as the mean of three independent experiments ± SEM as variability B H1581, KATO III and RT-112 cells were exposed to serial dilutions of Pem (25–400 nM) for 24 h and 48 h, and intracellular Ki67 levels were then evaluated by flow cytometry. The histograms represent the geo-mean of Ki67-BV421 MFI found in untreated (NT) and Pem-treated H1581, KATO III and RT-112 cells and are represented as mean of three independent experiments ± SEM. *p < 0.05; **p < 0.01; ***p ≤ 0.001 Student’s t test. Figure S2. Effect of Pem on the S and G2 phases of cell cycle. The histograms represent the count of PI-positive cells found in S- and G2-phase in untreated (NT, white) and Pem-treated (100 nM, grey) H1581, KATO III and RT-112 cells and are represented as mean of three independent experiments ± SEM. *p < 0.05; **, p < 0.01; ***p ≤ 0.001. Student’s t test. Figure S3. Effect of pemigatinib on ATP and HMGB1 release. A Extracellular HMGB1 release upon Pem treatment. The extracellular HMGB1 release was measured by means of an enzyme-linked immunosorbent assay (ELISA) kit (TECAN, Zürich, Switzerland), according to the manufacturer’s protocol. Histograms represent the mean values of three independent experiments ± SEM of HMGB1 released by untreated cells (NT, white) and Pem-treated cells (grey) at 24 and 48 h. ns, not significative. B Extracellular ATP release upon Pem treatment. The ATP release was measured by ENLITEN- [file 12967_2023_4450_MOESM1_ESM.docx]

**ADDITIONAL FILE 1**

Supplementary information for

**Targeting FGFRs by pemigatinib induces G1 phase cell cycle arrest, cellular stress and upregulation of tumor suppressor microRNAs**

Angelica Pace^1^*, Fabio Scirocchi^1^*, Chiara Napoletano^1@^, Ilaria Grazia Zizzari^1^, Agnese Po^2^, Francesca Megiorni^1^, Angela Asquino^1^, Paola Pontecorvi^1^, Hassan Rahimi^1^, Cinzia Marchese^1^, Elisabetta Ferretti^1^, Marianna Nuti^1^, Aurelia Rughetti^1^

**Supplementary Table 1.** FGFR expression was evaluated by Western blot and q-RT‒PCR experiments in H1581, KATO III, RT-112, RCC4plusVHL and DU-145 cancer cell lines employed as cell models of lung, gastric, bladder, renal and prostate cancer, respectively.


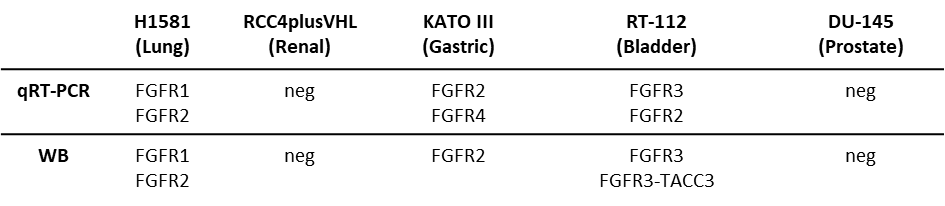


**H1581**

**KATO III**

**RT-112**

**A**

**B**

**24h**

**48h**

**48h**

**24h**

**Figure S1.** Serial dilution of Pem and proliferation analysis. A) For selection of the optimal drug concentration, H1581, KATO III and RT-112 cells were exposed to serial dilutions of Pem (3-1000 nM) for 24 h and 48h, and MTT proliferation assay was then performed. Data are reported as the mean of three independent experiments ± SEM as variability **B)** H1581, KATO III and RT-112 cells were exposed to serial dilutions of Pem (25-400 nM) for 24 h and 48h, and intracellular Ki67 levels were then evaluated by flow cytometry. The histograms represent the geo-mean of Ki67-BV421 MFI found in untreated (NT) and Pem-treated H1581, KATO III and RT-112 cells and are represented as mean of three independent experiments ± SEM. *, p<0,05; **, p< 0,01; ***, p≤ 0,001 Student’s t test.

**24h**

**48h**

**S phase**

**G2 phase**

**Figure S2.** Effect of Pem on the S and G2 phases of cell cycle. The histograms represent the count of PI-positive cells found in S- and G2-phase in untreated (NT, white) and Pem-treated (100 nM, grey) H1581, KATO III and RT-112 cells and are represented as mean of three independent experiments ± SEM. *, p<0,05; **, p< 0,01; ***, p≤ 0,001. Student’s t test.

**A**

**B**

**24h**

**Figure S3.** Effect of pemigatinib on ATP and HMGB1 release. A) Extracellular HMGB1 release upon Pem treatment. The extracellular HMGB1 release was measured by means of an enzyme-linked immunosorbent assay (ELISA) kit (TECAN, Zürich, Switzerland), according to the manufacturer’s protocol. Histograms represent the mean values of three independent experiments ± SEM of HMGB1 released by untreated cells (NT, white) and Pem-treated cells (grey) at 24 and 48 h. ns, not significative. B) Extracellular ATP release upon Pem treatment. The ATP release was measured by ENLITEN-Promega KIT as luminescence signals. The histograms represent the mean value of ATP moles of three independent experiments ± SEM by untreated cells (NT, white) and Pem-treated cells (Pem, grey) at 24 and 48 h. ns, not significative.

**RT-112**

**H1581**

**KATO III**

**A**

**B**

**C**

**Figure S4.** Effect of pemigatinib on miRNA target transcripts. mRNA expression of miRNAs targets in untreated (NT) and Pem-treated (100 nM) cancer cell lines. Histograms represent the mean values of three independent experiments ± SEM.

**C**
